# Analyzing Color, Surface Roughness, and Microhardness on the Unpolished and Polished Surfaces of Occlusal Splint Materials From Conventional and CAD–CAM Fabrication Methods

**DOI:** 10.1155/ijod/9002663

**Published:** 2026-03-02

**Authors:** Uthai Uma, Thanchanit Lertyingyos, Tharathip Lilitsuvan

**Affiliations:** ^1^ Department of Occlusion, Faculty of Dentistry, Chulalongkorn University, 34 Henri-Dunant Road, Wangmai, Pathumwan, Bangkok, 10330, Thailand, chula.ac.th; ^2^ Faculty of Dentistry, Chulalongkorn University, 34 Henri-Dunant Road, Wangmai, Pathumwan, Bangkok, 10330, Thailand, chula.ac.th

**Keywords:** 3D-printed, CAD–CAM, color, heat-cured, microhardness, milled, occlusal splint, self-cured, surface roughness

## Abstract

**Purpose:**

To evaluate the color, surface roughness, and microhardness of occlusal splints fabricated using conventional and CAD–CAM techniques and to compare the properties of the unpolished and polished surfaces.

**Materials and Methods:**

A total of 114 specimens (10 × 10 × 5 mm^3^) were prepared, including one group each of self‐cured (SC) and heat‐cured (HC) occlusal splints, along with two groups each of milled (ML‐A and ML‐B) and 3D‐printed (3D‐A and 3D‐B) splints. One side of each specimen was polished using a sequence of 600‐, 800‐, and 1000‐grit sandpaper, followed by pumice and tallow. The specimens were then tested for color parameters (*L* ^∗^, *a* ^∗^, *b* ^∗^), color change (∆*E*), surface roughness (Ra), and Vickers hardness number (VHN). Statistical analysis was performed using SPSS Version 29.0.

**Results:**

Significant variations were observed in color, surface roughness, and microhardness across the six materials (*p* < 0.001). For the unpolished surface, the highest and lowest color values were observed in ML‐B (75.10) and SC (60.55) for *L* ^∗^, SC (0.14) and 3D‐B (−2.19) for *a* ^∗^, and HC (2.47) and SC (−8.92) for *b* ^∗^. There was a significant difference in ∆*E* (*p* = 0.011), with ML‐A showing the lowest ∆*E*. The two with the lowest Ra values were ML‐A (0.085 µm) and ML‐B (0.117 µm). The highest microhardness values were found in ML‐B (19.74 VHN) and HC (18.85 VHN). Polishing significantly affected the properties (*p* < 0.05), with significant differences in *L* ^∗^ > *a* ^∗^ > *b* ^∗^, reduced Ra across all groups, and decreased the VHN in HC and 3D‐B groups.

**Conclusion:**

Occlusal splint materials fabricated using different techniques had significant differences in color, surface roughness, and microhardness. The ML‐B occlusal splint showed the best overall performance, with high *L* ^∗^, near‐zero *b* ^∗^, moderate *a* ^∗^, low surface roughness, and the highest microhardness. Polishing generally followed the color trend *L* ^∗^ > *a* ^∗^ > *b* ^∗^, reduced surface roughness in all groups, and decreased microhardness in HC and 3D‐B splints. Hence, some CAD–CAM materials can perform similarly to conventional splints, highlighting the importance of considering both material and fabrication methods to optimize clinical outcomes.

## 1. Introduction

An occlusal splint is an oral device available in various designs [1] to manage temporomandibular disorders [2, 3] and bruxism [4, 5]. These devices have been widely utilized for decades, serving as an effective noninvasive treatment option for conditions affecting the masticatory system [6]. Recently, there has been a resurgence of interest in occlusal splints, driven by advancements in their design, fabrication techniques, and expanded therapeutic applications [7]. This renewed focus highlights their evolving role in improving patient outcomes in clinical and research settings.

The fabrication of occlusal splints involves conventional techniques, such as self‐curing, heat‐curing, and vacuum forming, as well as digital techniques like milling and 3D printing [7]. In the digital era, a significant challenge lies in evaluating the efficiency and outcomes of digital methods compared with conventional ones. Digital workflows provide notable advantages, including greater accuracy [8], better fit [9], reduced need for adjustments [10], and streamlined design using specialized software [11]. Moreover, the dental materials used in digital techniques differ from those used in conventional methods [7], and these transformative materials should be thoroughly evaluated before being prescribed to patients who have different oral conditions.

The occlusal splint manufacturing method significantly influences their properties and clinical effectiveness [12–14]. A key characteristic of these splints is their ability to withstand the heavy forces exerted by patients’ teeth, requiring sufficient microhardness for durability. Additionally, occlusal splints have two distinct surfaces: the unpolished intaglio surface, which fits against the teeth, and the polished cameo surface, which contacts the opposing teeth [6]. These surfaces exhibit different roughness levels, potentially affecting bacterial adhesion. Last, the color of occlusal splints is typically clear to ensure esthetic acceptability and visibility in clinical use. Therefore, investigations of occlusal splint properties should emphasize material‐related characteristics that guide clinical selection, with particular attention to the differences between unpolished and polished surfaces—an aspect underrepresented in previous studies. Moreover, given the wide range of materials, brands, and fabrication techniques available, future research should evaluate a broader spectrum of products rather than relying on a single brand to represent an entire fabrication method.

Hence, the objective of this study was to evaluate and compare the color, surface roughness, and microhardness of the unpolished and polished surfaces of six different occlusal splint materials: one self‐cured (SC), one heat‐cured (HC), two milled (ML), and two 3D‐printed (3D) occlusal splint materials. The primary null hypothesis was that there would be no significant differences in color, surface roughness, and microhardness among the six occlusal splint materials. The secondary null hypothesis posited that there would be no significant differences in these properties between the unpolished and polished surfaces.

## 2. Materials and Methods

### 2.1. Sample Size

**Figure 1:**
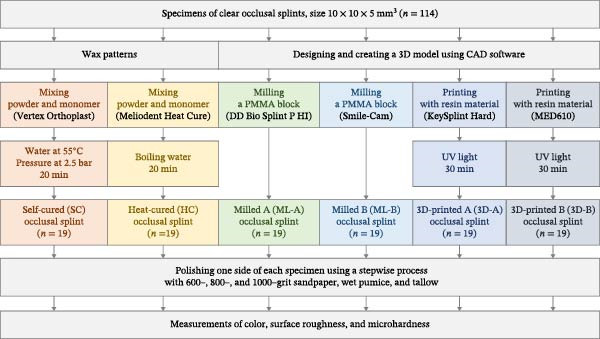
The study design included 114 specimens across all groups: self‐cured (SC), heat‐cured (HC), milled A (ML‐A), milled B (ML‐B), 3D‐printed A (3D‐A), and 3D‐printed B (3D‐B). Each group comprised 19 specimens, which were subjected to subsequent measurements of color, surface roughness, and microhardness.

This study assessed the color, surface roughness, and microhardness of occlusal splint materials produced using various fabrication techniques. It analyzed the mean differences across six groups of independent specimens: one SC group, one HC group, two ML groups, and two 3D‐printed groups. The sample size was calculated using 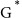
Power software Version 3.1.9.7, focusing on the *F* test family and one‐way ANOVA. According to Guimaraes et al. [13], the mean surface roughness values were SC at 0.08 ± 0.05 µm, HC at 0.09 ± 0.06 µm, HC at 0.07 ± 0.05 µm, ML at 0.10 ± 0.02 µm, and 3D‐printed at 0.15 ± 0.08 µm. With 10 specimens per group, a power of 0.80, and a significance level of 0.05, the total required sample size for this study was determined to be 19 specimens per group, requiring 114 specimens for the six occlusal splint material groups (Figure 1).

### 2.2. Specimen Preparation

In the SC group, 19 specimens were created using 10 × 10 × 5 mm^3^ rectangular wax patterns, which were embedded in a silicone mold. The wax was then removed and replaced with a mixture of clear acrylic powder and monomer (Vertex Orthoplast, Vertex Dental, Soesterberg, Netherlands) in a 2.1 g to 1 mL powder‐to‐liquid ratio. These specimens were placed in a pressure pot (Vertex MultiCure, Vertex Dental, Soesterberg, Netherlands) with water at 55°C and a pressure of 2.5 bars for 20 min.

For the HC group, 19 specimens were also made from 10 × 10 × 5 mm^3^ rectangular wax patterns placed in flasks. The wax was melted out and replaced with a mixture of clear acrylic resin powder and monomer (Meliodent Heat Cure, Kulzer, Hanau, Germany) in a 35 g to 14 mL powder‐to‐liquid ratio. The flasks were submerged in boiling water for 20 min to ensure complete polymerization, following the manufacturer’s instructions.

The ML groups consisted of two subgroups: ML‐A and ML‐B. In the ML‐A group, the specimens were designed as cuboid blocks measuring 10 × 10 × 5 mm^3^ using CAD software (Blender 2.79, Blender Institute BV, Amsterdam, Netherlands). A clear polymethyl methacrylate (PMMA) block (DD Bio Splint P HI, Dental Direkt GmbH, Spenge, Germany) was ML to the target dimensions using a 4‐axis milling machine (Arum Dental Mill 4X‐450, Arum Europe GmbH, Schwalbach am Taunus, Germany) for 19 ML specimens. For the ML‐B group, a different clear PMMA block (Smile‐Cam PMMA Calcinable, Pressing Dental SRL, Dogana, San Marino Republic) was ML to 10 × 10 × 5 mm^3^ using a 5‐axis milling machine (vnf S2, vhf camfacture AG, Ammerbuch, Germany) for 19 ML specimens.

The 3D groups included two subgroups: 3D‐A and 3D‐B. The 3D‐A specimens were designed and created as 10 × 10 × 5 mm^3^ blocks using the same CAD software as the ML groups. These were printed with biocompatible materials designed for clear occlusal splints (KeySplint Hard Clear, HeyGears, Guangzhou, China) on a 3D printer (UltraCraft A2D, HeyGears, Guangzhou, China) using the manufacturer’s recommended settings with 0° orientation and 0.1‐mm layer thickness. This 3D printer utilizes digital light processing (DLP) technology, curing the resin layer by layer with projected light. In the 3D‐B group, the specimens were printed with a biocompatible clear resin (MED610, Biocompatible Clear, Stratasys, Minnesota, USA) on a 3D printer (Stratasys Objet260 Dental, Stratasys, Minnesota, USA) following the standard parameters, including 0° orientation and 0.1‐mm layer thickness. This printer employs PolyJet technology, which jets multiple photopolymer materials in ultrafine layers that are immediately cured by UV light. After fabrication of the 3D specimens (3D‐A and 3D‐B groups), postprocessing was performed. The specimens were cleaned in an ultrasonic bath with isopropyl alcohol to remove uncured resin, rinsed with water, and then postcured under UV light using a curing unit (LC‐3DPrint Box, NextDent, Vertex‐Dental, Soesterberg, Netherlands) for 30 min to enhance polymerization and ensure material stability.

For SC and HC specimens, the outer surfaces that did not contact the silicone mold were designated as unpolished to represent the raw material after polymerization, while the opposite surfaces in contact with the mold were assigned as polished. ML‐A and ML‐B specimens were randomly assigned to have either unpolished or polished surfaces. For 3D‐A and 3D‐B specimens, the support structures were removed using cutting pliers; the surfaces that had contacted the supports were designated as polished, and the others as unpolished. One side of each specimen was polished using a stepwise process with 600‐, 800‐, and 1000‐grit waterproof abrasive paper (TOA Paint, Samutprakan, Thailand) on an automatic polisher (EcoMet 30, Buehler, USA) at 500 rpm with 5 N force for 1 min per step. Final finishing was performed with wet pumice (Lab pumice‐medium grit, Shanghai Dental Material, Bangkok, Thailand) and tallow (Shanghai Dental Material, Bangkok, Thailand) on a polishing machine (Polix 905, Silfradent, St. Sofia, Italy) at 1400 rpm for 1 min to achieve a high shine. The other sides were left untreated (Figure 2A). After preparation, all specimens were rinsed with water and stored in a dry container for 7 days to simulate transportation time from the dental laboratory to the clinic.

Figure 2Specimen preparation and measurement protocols: (A) one side was polished, while the other remained unpolished, (B) color measurements conducted three times near the specimen center, (C) surface roughness measured three times along three 5‐mm lines using a contact stylus, and (D) microhardness measured at three points with pyramid‐shaped indentations.

**Figure:**
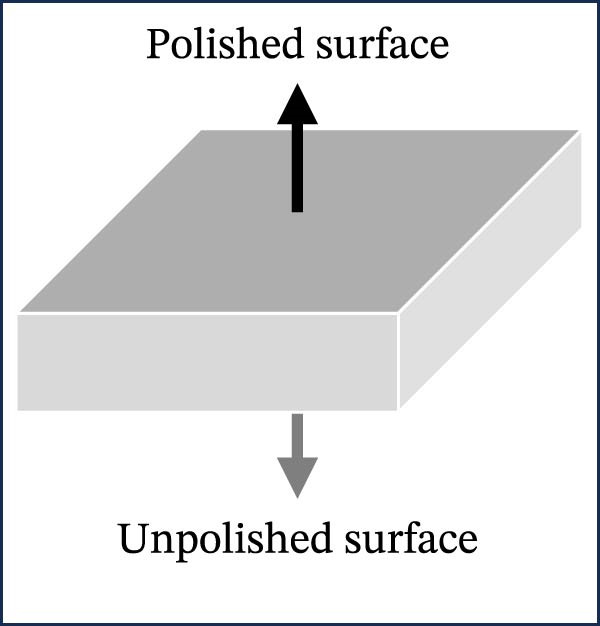
(A)

**Figure:**
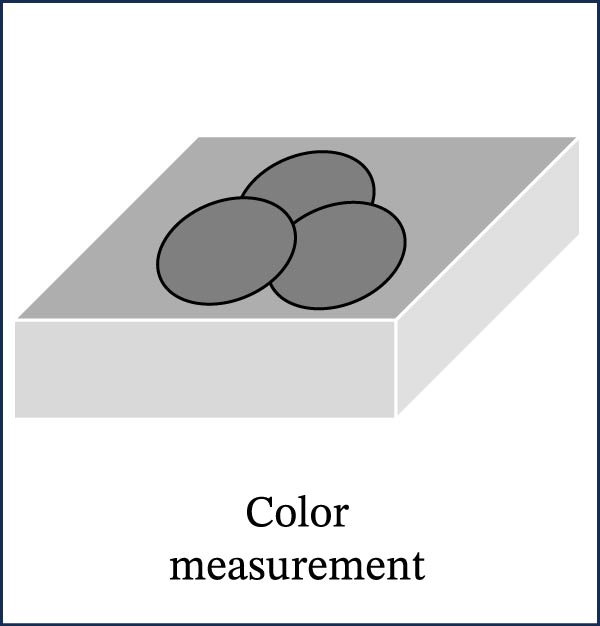
(B)

**Figure:**
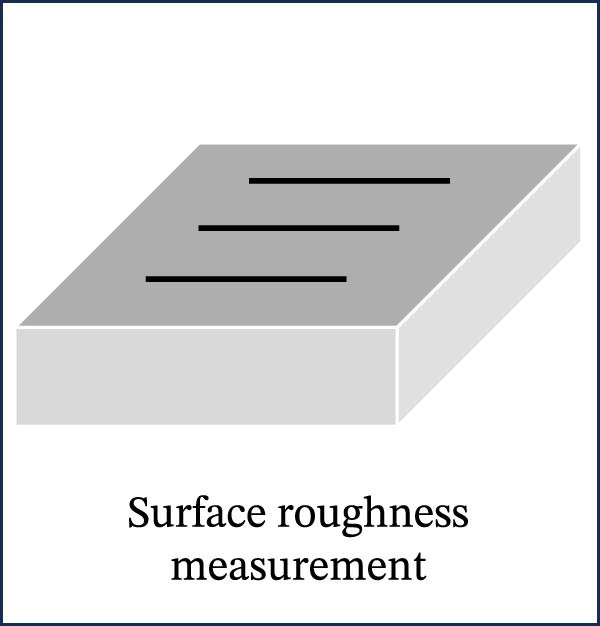
(C)

**Figure:**
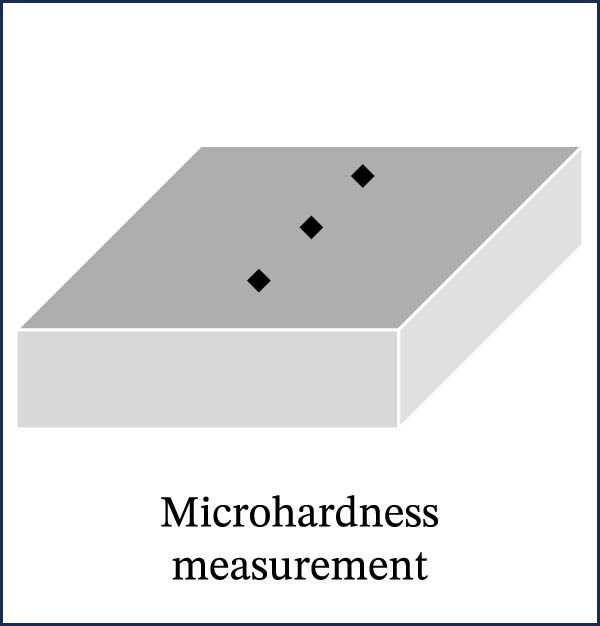
(D)

### 2.3. Color Measurement

A total of 114 specimens were analyzed, with 19 from each group: SC, HC, ML‐A, ML‐B, 3D‐A, and 3D‐B. Color measurements were conducted using a spectrocolorimeter (Ultrascan PRO, Hunter Lab, Virginia, USA) in reflectance mode, utilizing a small area view with a 7 mm port size and a 4 mm measurement area. Each specimen’s color was measured three times at the specimen’s central area (Figure 2B), and mean values were recorded for *L* ^∗^ (lightness, where *L* ^∗^ = 0 represents black and *L* ^∗^ = 100 represents white), *a* ^∗^ (red‐greenness, with negative values indicating green and positive values indicating red), and *b* ^∗^ (yellow‐blueness, where negative values indicated blue and positive values indicated yellow). The *L* ^∗^, *a* ^∗^, and *b* ^∗^ values were individually recorded to identify specific trends in color change along each axis of the 3D color model [15], rather than focusing solely on the overall color outcome. This systematic approach ensured accurate and reliable color assessments across the specimen groups. Moreover, the overall color change (∆*E*) after polishing was calculated using the formula, ∆*E* = [(*L*_2_^∗^ − *L*_1_^∗^)^2^ + (*a*_2_^∗^ − *a*_1_^∗^)^2^ + (*b*_2_^∗^ − *b*_1_^∗^)^2^]^1/2^, where *L*_2_^∗^, *a*_2_^∗^, and *b*_2_^∗^ were the postpolished color values, and *L*_1_^∗^, *a*_1_^∗^, and *b*_1_^∗^ were the prepolished color values [11, 16].

### 2.4. Surface Roughness Measurement

Surface roughness was assessed using a contact surface roughness tester (Talyscan 150, Taylor Hobson, Leicester, England). Each specimen was tested three times at randomly selected areas around the center, with a contact length of 5 mm for each measurement (Figure 2C). The mean line surface roughness (Ra) value was calculated for further analysis. Ra, which represents the arithmetic average of surface height deviations, was chosen over the area surface roughness (Sa) value because it is the most widely used and standardized parameter for contact profilometry. In addition, Ra provides reliable and reproducible results for evaluating dental materials, while Sa is more appropriate for 3D areal measurements obtained from noncontact methods.

Additionally, one specimen from each group, totaling six specimens with 12 surfaces (both polished and unpolished), was randomly selected and sputter‐coated with a 99.99% gold layer using a sputter coater (Gold Coater, JFC‐1200, Jeol, Massachusetts, USA). This preparation enabled the examination of surface roughness morphology using a scanning electron microscope (SEM) (Quanta 250 FEG, FEI, Oregon, USA) at a magnification of 1000× in high vacuum mode.

### 2.5. Microhardness Measurement

Finally, microhardness testing was performed using a microhardness tester equipped with a Vickers indenter (FM‐810, Future‐Tech, Kawasaki, Japan), applying a load of 1.96 N for 15 s [17]. The diagonals of the resulting pyramidal indentations were measured optically by an operator using the machine’s eyepiece. The Vickers hardness number (VHN) was calculated using the formula VHN = 0.1891(*F*/*d* ^2^), where *F* is the force in newtons and *d* is the average length of the two diagonals in millimeters. Each specimen was measured three times (Figure 2D), and the mean value was used for further analysis.

### 2.6. Data Collection and Analysis

To ensure consistency in data collection, three examiners were designated as follows: one for color measurement, one for the surface roughness test, and another for microhardness assessment. Each examiner was responsible for testing and recording all data for every specimen. Statistical analysis was conducted using IBM SPSS Statistics for Windows, Version 29.0 (IBM Corporation, Armonk, New York, USA). The Shapiro–Wilk test was utilized to evaluate the data distribution. One‐way ANOVA and the Kruskal–Wallis test were employed to compare the mean differences among the six groups, while the paired *t*‐test and the Wilcoxon signed‐rank test assessed the mean differences between the polished and unpolished specimens. Two‐way ANOVA was conducted to evaluate the effects of material type, polishing, and their interaction. A *p*‐value of 0.05 was deemed significant at a 95% confidence level.

## 3. Results

### 3.1. Color Analysis

**Figure 3:**
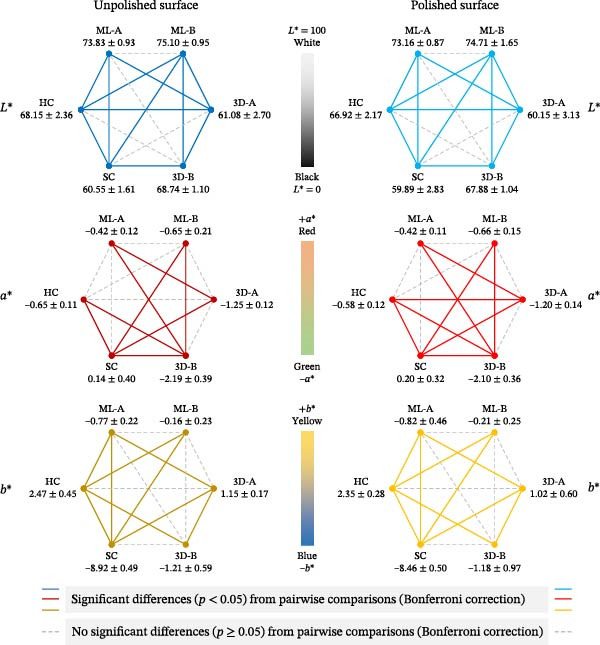
Color value analysis for mean differences and pairwise comparisons. *L* ^∗^ (lightness: 0 = black, 100 = white), *a* ^∗^ (red‐greenness: positive *a* ^∗^ = red and negative *a* ^∗^ = green), and *b* ^∗^ (yellow‐blueness: positive *b* ^∗^ = yellow and negative *b* ^∗^ = blue) was performed on the unpolished and polished surfaces. The Kruskal–Wallis test comparing the means of the six materials indicated significant differences in each color’s value (*p* < 0.001). Pairwise comparisons using the Bonferroni correction revealed that the solid lines for blue, red, and yellow showed significant differences (*p* < 0.05), while the dashed gray line indicated no significant differences (*p* ≥ 0.05).

Because the Shapiro–Wilk test showed a significant deviation from normality (*p* < 0.05), nonparametric statistical analyses were applied to all color measurement parameters (Table 1 and Figure 3). Based on the Kruskal–Wallis test comparing unpolished and polished surfaces of six occlusal splint materials (Table 1), all color parameters (*L* ^∗^, *a* ^∗^, and *b* ^∗^) showed significant differences among groups (*p* < 0.001), indicating that the type of occlusal splint material influenced each color parameter. Furthermore, analysis of the differences between polished and unpolished surfaces, representing the effect of polishing, also revealed significant differences for all color parameters (*L* ^∗^: *p* = 0.018; *a* ^∗^: *p* = 0.007; *b* ^∗^: *p* = 0.002). The ML‐B splints exhibited the highest *L* ^∗^ values (indicating white), while the SC splints had the lowest *L* ^∗^ values (indicating black). The SC splints also had the highest *a* ^∗^ values (indicating red), while the 3D‐B splints displayed the lowest *a* ^∗^ values (indicating green). For the *b* ^∗^ values, the HC splints had the highest values (indicating yellow), whereas the 3D‐B splints showed the lowest *b* ^∗^ values (indicating blue). The overall color change (∆*E*), calculated from the differences between polished and unpolished occlusal splints, is presented in Table 1. The Kruskal–Wallis test revealed a significant difference in ∆*E* values among the six groups (*p* = 0.011), indicating that the type of occlusal splint material influenced the overall color change. Among the materials tested, ML‐A exhibited the lowest ∆*E* value, whereas HC showed the highest ∆*E* value.

**Table 1:** Results of the Kruskal–Wallis test comparing color parameters among six occlusal splint materials at unpolished and polished surfaces, including the effect of polishing (difference between polished and unpolished surfaces) and overall color change.

Variables	Occlusal splint materials (median ± IQR)						*p*‐Values^1^
	SC	HC	ML‐A	ML‐B	3D‐A	3D‐B	
Color (*L* ^∗^), (lightness: 0 = black, 100 = white)							
Unpolished (*L*_1_^∗^)	60.55 ± 1.61^a^	68.15 ± 2.36^b^	73.83 ± 0.93^c,d^	75.10 ± 0.95^c,d^	61.08 ± 2.70^a^	68.74 ± 1.10^c^	<0.001 ^∗^
Polished (*L*_2_^∗^)	59.89 ± 2.83^a^	66.92 ± 2.17^b^	73.16 ± 0.87^c^	74.71 ± 1.65^c^	60.15 ± 3.13^a^	67.88 ± 1.04^b,c^	<0.001 ^∗^
Difference (*L*_2_^∗^ − *L*_1_^∗^)	−0.58 ± 1.28^a,b^	−1.28 ± 0.74^a^	−0.52 ± 0.66^b^	−0.53 ± 1.15^a,b^	−0.85 ± 1.60^a,b^	−0.84 ± 0.91^a,b^	0.018 ^∗^
Color (*a* ^∗^), (red‐greenness: *a* ^∗^ = red, −*a* ^∗^ = green)							
Unpolished (*a*_1_^∗^)	0.14 ± 0.40^d^	−0.65 ± 0.11^b,c^	−0.42 ± 0.12^c,d^	−0.65 ± 0.21^b,c^	−1.25 ± 0.12^a,b^	−2.19 ± 0.39^a^	<0.001 ^∗^
Polished (*a*_2_^∗^)	0.20 ± 0.32^e^	−0.58 ± 0.12^c,d^	−0.42 ± 0.11^d,e^	−0.66 ± 0.15^b,c^	−1.20 ± 0.14^a,b^	−2.10 ± 0.36^a^	<0.001 ^∗^
Difference (*a*_2_^∗^ − *a*_1_^∗^)	−0.06 ± 0.25^a^	0.05 ± 0.06^a^	−0.02 ± 0.04^a^	−0.01 ± 0.13^a^	0.04 ± 0.07^a^	0.09 ± 0.18^a^	0.007 ^∗^
Color (*b* ^∗^), (yellow‐blueness: *b* ^∗^ = yellow, −*b* ^∗^ = blue)							
Unpolished (*b*_1_^∗^)	−8.92 ± 0.49^a^	2.47 ± 0.45^d^	−0.77 ± 0.22^b^	−0.16 ± 0.23^b,c^	1.15 ± 0.17^c,d^	−1.21 ± 0.59^a,b^	<0.001 ^∗^
Polished (*b*_2_^∗^)	−8.46 ± 0.50^a^	2.35 ± 0.28^d^	−0.82 ± 0.46^b^	−0.21 ± 0.25^b,c^	1.02 ± 0.60^c,d^	−1.18 ± 0.97^a,b^	<0.001 ^∗^
Difference (*b*_2_^∗^ − *b*_1_^∗^)	0.47 ± 0.54^b^	−0.05 ± 0.19^a^	−0.04 ± 0.13^a^	0.02 ± 0.38^a,b^	−0.09 ± 0.46^a^	0.11 ± 0.38^a^	0.002 ^∗^
Overall color change (∆*E*), ∆ *E* = [(*L*_2_^∗^‐ *L*_1_^∗^)^2^ + (*a*_2_^∗^‐ *a*_1_^∗^)^2^ + (*b*_2_^∗^‐ *b*_1_^∗^)^2^]^1/2^							
∆ *E*	1.06 ± 0.93^a,b^	1.29 ± 0.74^b^	0.55 ± 0.23^a^	0.91 ± 1.32^a,b^	0.92 ± 1.14^a,b^	0.98 ± 0.67^a,b^	0.011 ^∗^

*Note:* *L* ^∗^,*a* ^∗^, and *b* ^∗^ = color parameters. 3D‐A, 3D‐printed A; 3D‐B, 3D‐printed B. ^a, b, c, d, e^ pairwise comparison (Bonferroni correction), different superscript letters denote statistical differences (*p* < 0.05).

Abbreviations: HC, heat‐cured; IQR, interquartile range; ML‐A, milled A; ML‐B, milled B; SC, self‐cured.

^1^Kruskal–Wallis test.

^∗^denotes statistical differences (*p* < 0.05).

Pairwise comparisons, with significant differences indicated by solid lines, are also shown in Figure 3. Several significant differences (*p* < 0.05) were observed across material pairs for *L* ^∗^, *a* ^∗^, and *b* ^∗^ values. Notably, the overall patterns of pairwise differences remained consistent when comparing unpolished and polished surfaces for each color parameter. The only exception was the pair of HC and 3D‐A materials, which showed a significant difference in *a* ^∗^ value between the unpolished and polished surfaces.

**Figure 4:**
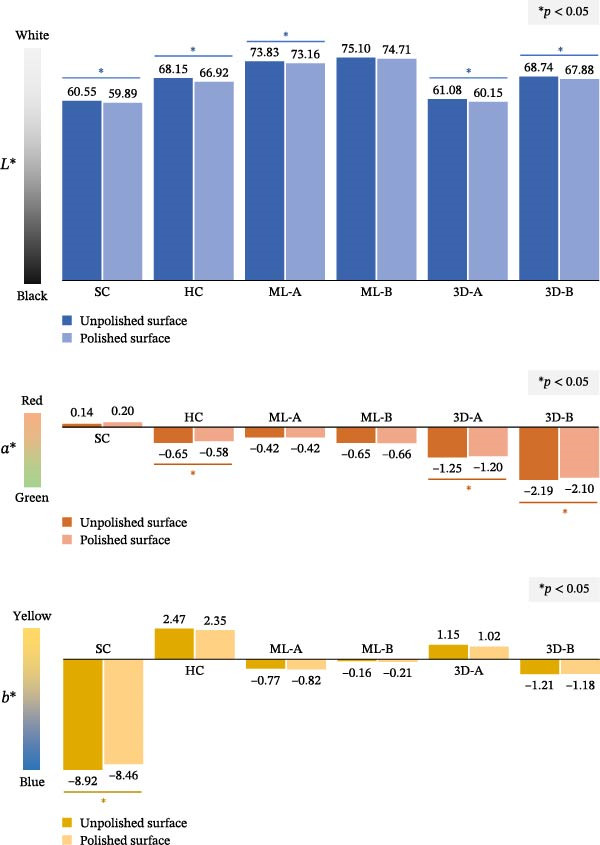
Color analysis comparing the unpolished and polished surfaces of the six occlusal splint materials. The comparisons were conducted using the Wilcoxon signed‐rank test, with statistical significance indicated by an asterisk (*p* < 0.05). The *L* ^∗^ value represents lightness, where 0 indicates black and 100 indicates white; the *a* ^∗^ value reflects red‐green balance, with positive values indicating red and negative values indicating green; and the *b* ^∗^ value corresponds to yellow‐blue balance, where positive values indicate yellow and negative values indicate blue.

To compare color values between the unpolished and polished surfaces, the Wilcoxon signed‐rank test was applied (Figure 4). The results demonstrated that the polished surfaces had significantly lower *L* ^∗^ values (indicating darker shades) than the unpolished surfaces in all groups, except for the ML‐B group (*p* < 0.05). The *a* ^∗^ values revealed significant differences (*p* < 0.05) in the HC, 3D‐A, and 3D‐B groups, with polished surfaces showing higher *a* ^∗^ values (indicating less green). For *b* ^∗^ values, a significant difference (*p* < 0.05) was found only in the SC group, where the polished surfaces had higher *b* ^∗^ values (indicating less blue).

### 3.2. Surface Roughness Analysis

Table 2 presents the surface roughness of occlusal splints, which was significantly affected by material type, polishing, and their interaction (*p* < 0.001). The statistical model explained 85.5% of the variance, indicating a strong model fit. Moreover, Table 3 displays the mean surface roughness values for the various occlusal splints. For the unpolished surfaces, significant differences in roughness were observed among the six materials (*p* < 0.001), as determined by one‐way ANOVA. The 3D‐A splint exhibited the highest surface roughness (0.718 ± 0.042 µm), while the ML‐A splint had the lowest (0.085 ± 0.007 µm), followed by the ML‐B splint. However, post hoc Tukey tests revealed significant differences (*p* < 0.05) among the SC and 3D‐A, the HC and 3D‐B, and the ML‐A and ML‐B groups, as indicated by different superscript letters in Table 1. For the polished surfaces, most materials exhibited similar surface roughness values, with the exception of the ML‐B group, which had the lowest roughness (0.035 ± 0.001 µm) and differed significantly from the other groups (*p* < 0.05). Figure 5 compares the surface roughness of the unpolished and polished surfaces, indicating that the polishing process significantly reduced surface roughness for all materials (*p* < 0.05).

Figure 5Surface roughness and microhardness analyses comparing the unpolished and polished surfaces of the six occlusal splint materials. Statistical comparisons were performed using paired *t*‐tests, with significant differences indicated by an asterisk (*p* < 0.05). (A) Surface roughness analysis and (B) microhardness analysis.

**Figure:**
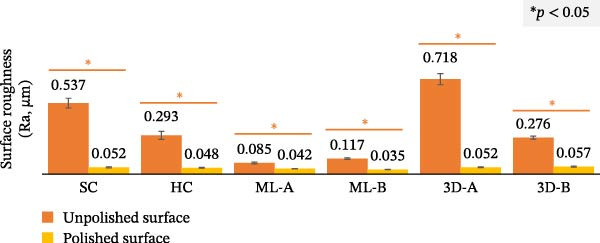
(A)

**Figure:**
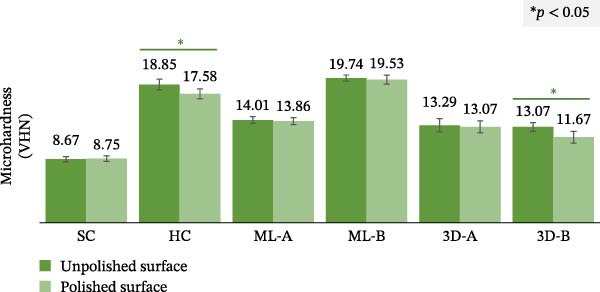
(B)

**Table 2:** Results of two‐way ANOVA assessing the effects of different material types, polishing of occlusal splint, and their interaction on color, surface roughness, and microhardness.

Variables	Source of variation	Type III SS	df	MS	*F*	*p*‐Value
Surface roughness	Materials	2.35	5	0.47	68.77	<0.001 ^∗^
	Polishing	4.26	1	4.26	623.79	<0.001 ^∗^
	Materials × polishing	2.12	5	0.42	62.17	<0.001 ^∗^
	*R* ^2^ = 0.855 (adjusted *R* ^2^ = 0.848)					

Microhardness	Materials	3044.84	5	608.97	1552.06	<0.001 ^∗^
	Polishing	15.73	1	15.73	40.08	<0.001 ^∗^
	Materials × polishing	19.23	5	3.85	9.80	<0.001 ^∗^
	*R* ^2^ = 0.973 (adjusted *R* ^2^ = 0.972)					

*Note:* Adjusted *R*², adjusted coefficient of determination; *F*, *F*‐value; *R*², coefficient of determination.

Abbreviations: df, degree of freedom; MS, mean square; SS, sum of squares.

^∗^Significant difference (*p* < 0.05) from the two‐way ANOVA test.

**Table 3:** The one‐way ANOVA results for surface roughness and microhardness of the six occlusal splints are presented as means and standard deviations (SDs).

Variables	Occlusal splint materials (mean ± SD) and (95% CI)						
	SC	HC	ML‐A	ML‐B	3D‐A	3D‐B	*p*‐Values^1^
Surface roughness (Ra [µm])							
Unpolished surface	0.537 ± 0.037^a^	0.293 ± 0.030^b^	0.085 ± 0.007^c^	0.117 ± 0.006^c^	0.718 ± 0.042^a^	0.276 ± 0.011^b^	<0.001 ^∗^
	[0.460, 0.614]	[0.230, 0.356]	[0.070, 0.099]	[0.104, 0.131]	[0.529, 0.707]	[0.252, 0.301]	
Polished surface	0.052 ± 0.004^a^	0.048 ± 0.003^a^	0.042 ± 0.001^a,b^	0.035 ± 0.001^b^	0.052 ± 0.003^a^	0.057 ± 0.004^a^	<0.001 ^∗^
	[0.045, 0.60]	[0.040, 0.055]	[0.039, 0.045]	[0.033, 0.036]	[0.046, 0.059]	[0.048, 0.066]	
Microhardness (VNH)							
Unpolished surface	8.67 ± 0.36^a^	18.85 ± 0.74^b^	14.01 ± 0.46^c^	19.74 ± 0.41^d^	13.29 ± 0.90^e^	13.07 ± 0.59^e^	<0.001 ^∗^
	[8.49, 8.84]	[18.49, 19.20]	[13.78, 14.23]	[19.55, 19.94]	[12.86, 13.72]	[12.78, 13.35]	
Polished surface	8.75 ± 0.37^a^	17.58 ± 0.68^b^	13.86 ± 0.47^c^	19.53 ± 0.61^d^	13.07 ± 0.81^e^	11.67 ± 0.81^f^	<0.001 ^∗^
	[8.58, 8.93]	[17.25, 17.90]	[13.63, 14.09]	[19.24, 19.82]	[12.69, 13.46]	[11.28, 12.06]	

*Note:* 3D‐A, 3D‐printed A; 3D‐B, 3D‐printed B; 95% CI, the range within which 95% are confident that the true value lies. ^a, b, c, d, e, f^ post hoc Tukey test, different superscript letters denote statistical differences (*p* < 0.05).

Abbreviations: HC, heat‐cured; ML‐A, milled A; ML‐B, milled B; SC, self‐cured; SD, standard deviations.

^1^one‐way ANOVA test.

^∗^denotes statistical differences (*p* < 0.05).

Additionally, Figure 6 comprises the SEM images of the surface morphology of the materials, illustrating the texture differences between polished and unpolished surfaces and highlighting the changes caused by the polishing process. An unpolished SC splint had a rough surface with visible grooves, indicating a coarse texture. The polished surface was smoother, with fine lines and minor surface patterns. The unpolished HC splint showed pronounced striations and a rough texture, while polishing significantly smoothed the surface, though faint striations remained. The unpolished ML‐A splint exhibited moderate roughness with linear features, but polishing resulted in a very smooth, uniform surface. The unpolished ML‐B splint was relatively smoother than the others, with its polished surface retaining slight texture. The unpolished 3D‐A splint was highly irregular and porous, but polishing showed notable improvement, though some irregularities remained. Last, the unpolished 3D‐B splint had a very rough, complex morphology, and while polishing improved the surface, some texture persisted.

Figure 6Representative SEM images at 1000× magnification showing the surface morphology of occlusal splints. Unpolished surfaces: (A) SC—rough and uneven, (B) HC—longitudinal grooves and moderate roughness, (C) ML‐A—smooth with faint milling marks, (D) ML‐B—slightly smoother that ML‐A, (E) 3D‐A—intricate and uneven morphology with significant grooves and ridges, and (F) 3D‐B—rough and highly irregular surface with voids. Polished surfaces: (G) SC—smoother but with micro‐scratches, (H) HC—uniform and minor abrasions, (I) ML‐A, (J) ML‐B—minimal imperfections, (K) 3D‐A, and (L) 3D‐B—reduced roughness but some irregularities persist.

**Figure:**
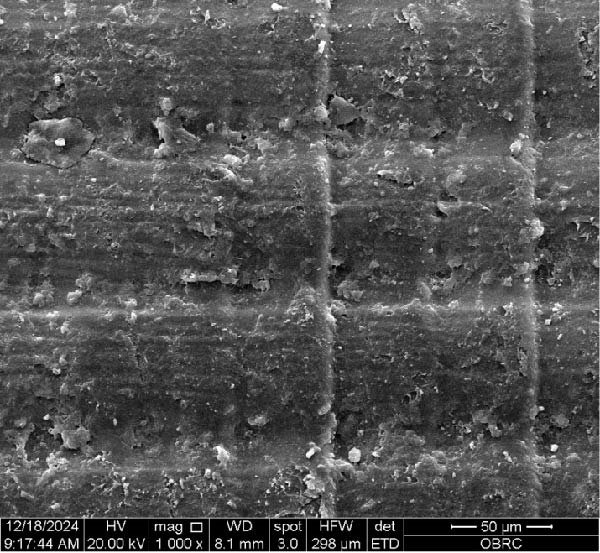
(A)

**Figure:**
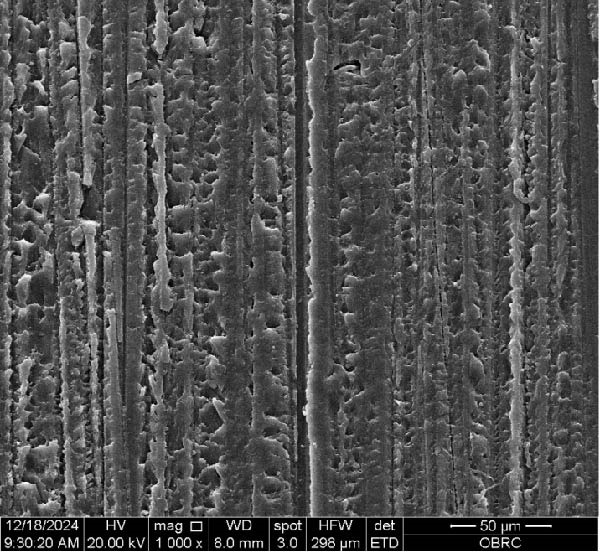
(B)

**Figure:**
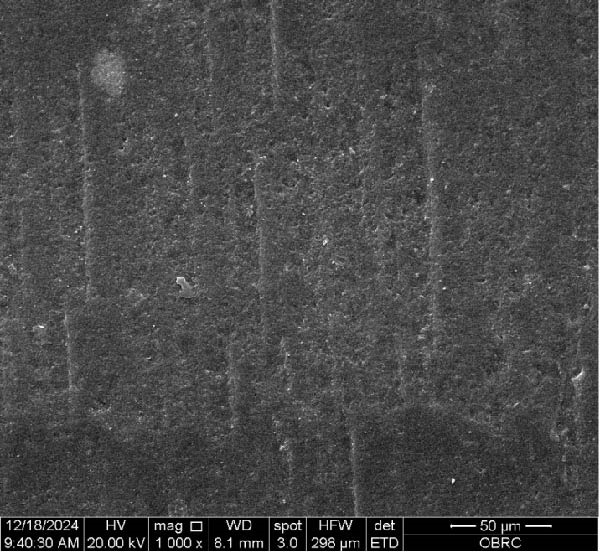
(C)

**Figure:**
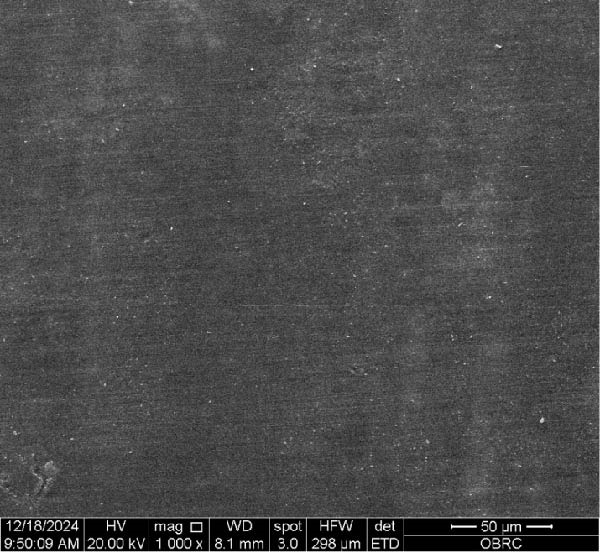
(D)

**Figure:**
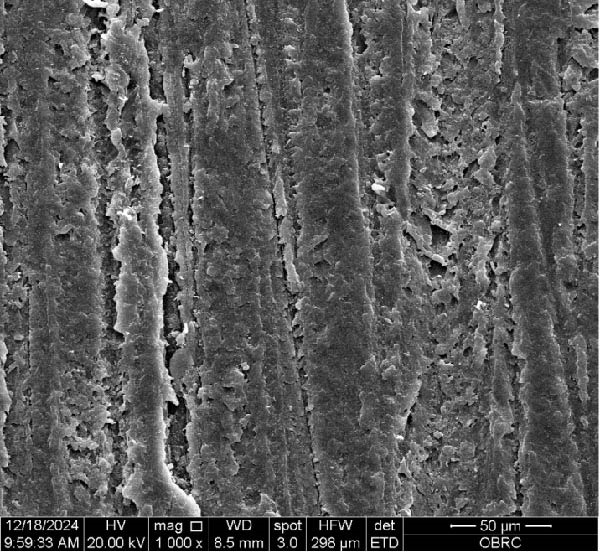
(E)

**Figure:**
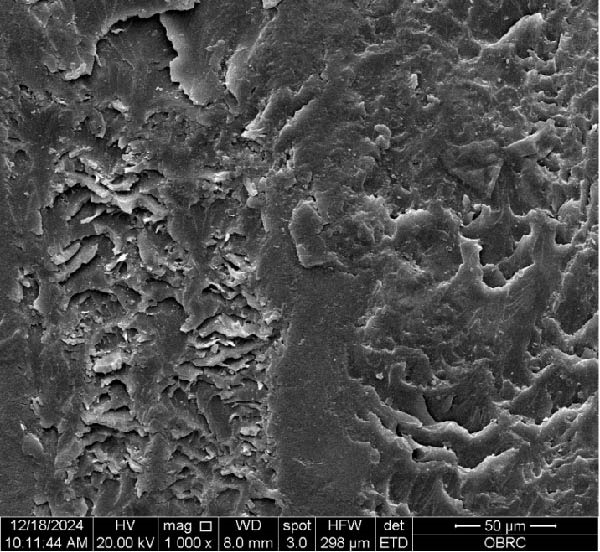
(F)

**Figure:**
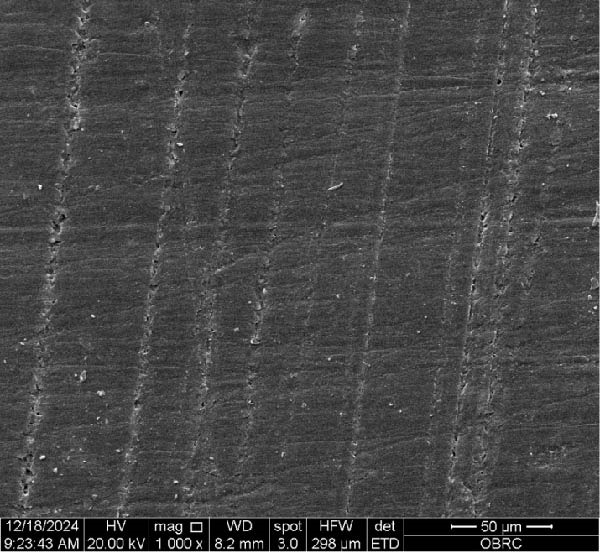
(G)

**Figure:**
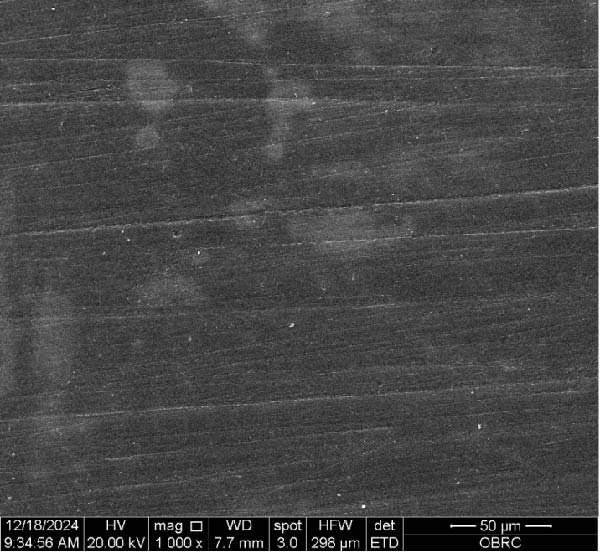
(H)

**Figure:**
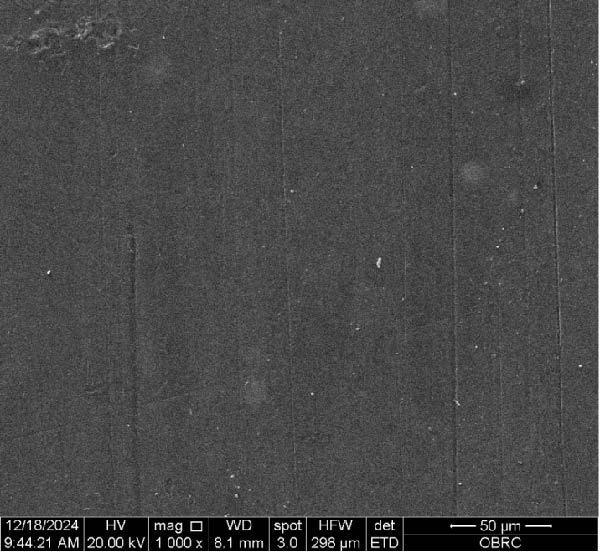
(I)

**Figure:**
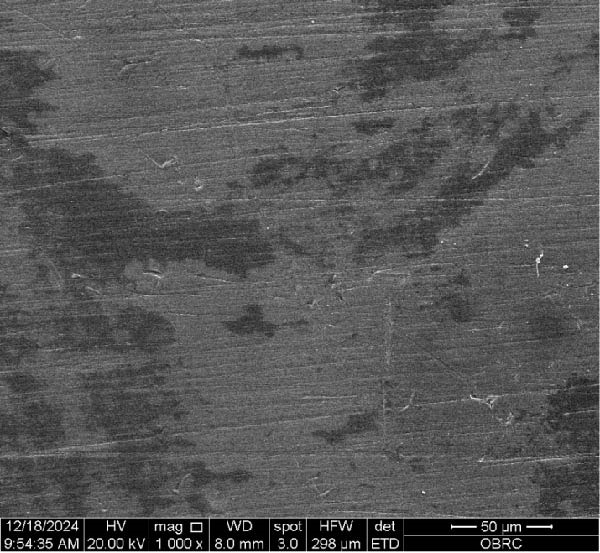
(J)

**Figure:**
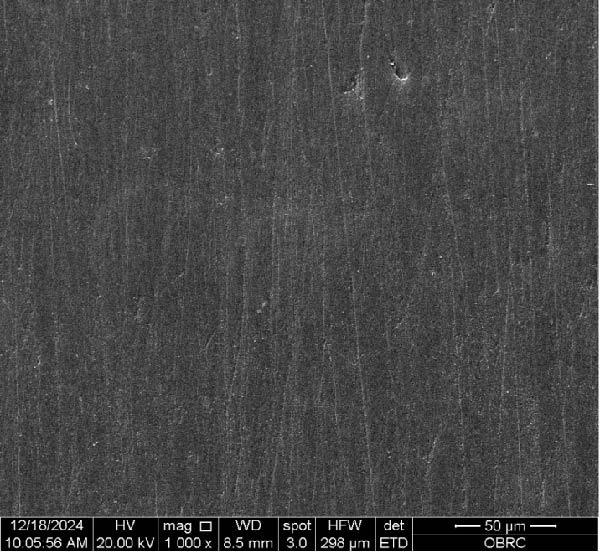
(K)

**Figure:**
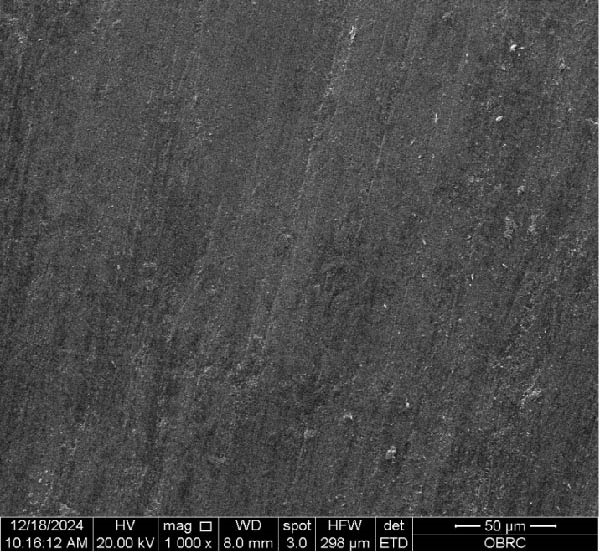
(L)

### 3.3. Microhardness Analysis

Table 2 also shows that the microhardness of occlusal splints was significantly affected by material type, polishing, and their interaction (*p* < 0.001). The statistical model explained 97.3% of the variance. Table 3 presents the mean microhardness values, indicating significant differences among the six materials for the unpolished and polished surfaces (*p* < 0.001), as determined by one‐way ANOVA. The post hoc Tukey test revealed statistically significant differences between all material pairs (*p* < 0.05), except for the unpolished 3D‐A and 3D‐B groups, as indicated by the shared superscript in Table 1. ML‐B demonstrated the highest microhardness, with values of 19.74 ± 0.41 VHN (unpolished) and 19.53 ± 0.61 VHN (polished), followed by the HC and ML‐A splints. In contrast, the SC splints had the lowest microhardness, with values of 8.67 ± 0.36 VHN (unpolished) and 8.75 ± 0.37 VHN (polished), ~2.5 times lower than that of ML‐B.

As shown in Figure 5, comparisons between the unpolished and polished surfaces of each material revealed that polishing slightly reduced microhardness in most materials, except for the SC splint, which showed a slight increase. However, significant differences in microhardness were observed only in the HC and 3D‐B groups (*p* < 0.05). In contrast, the other materials exhibited no significant differences (*p* > 0.05).

## 4. Discussion

The current study examined the properties of six different occlusal splint materials and identified significant differences in color, surface roughness, and microhardness. Consequently, the primary null hypothesis, which stated that there would be no significant differences in these properties, was not accepted, confirming that different materials exhibited distinct characteristics. These findings are consistent with previous studies, such as Hickl et al. [18] on surface roughness and Gibreel et al. [17] on microhardness, both of which reported notable variations in the properties of occlusal splint materials. Comparing the properties of the unpolished and polished surfaces, the polishing of occlusal splints significantly changed the color and microhardness in some groups and the surface roughness in all groups. Therefore, the secondary null hypothesis, which stated that there would be no significant differences in these properties between the two surface sides, was also not accepted for color, surface roughness, and microhardness.

### 4.1. Color

The occlusal splint materials in this study showed *L* ^∗^ values of 59.89–75.10 (white appearance), *a* ^∗^ values of –2.19 to 0.20 (slightly greenish rather than red), and *b* ^∗^ values of –8.92 to 2.47 (bluish rather than yellow). These values reflect the optical properties of clear materials. Considering the 3D color model rather than individual parameters [15], the overall coordinates represent the appearance of each specimen. For example, the SC material appeared light navy (*L* ^∗^ ~60, *a* ^∗^ ~0.2, and *b* ^∗^ ~–8.5), whereas the ML‐A and ML‐B splints had near‐zero *a* ^∗^ and *b* ^∗^ values, appearing clearer. However, the detailed information from each parameter remains important for critical evaluation and meaningful interpretation. This study also revealed differences in specific color parameters. These findings indicate that although occlusal splints are labeled as “clear,” their final color after fabrication may vary depending on the material and fabrication method.

The polished surface of an occlusal splint is visible to both patients and dentists [19]. Polishing significantly reduced whiteness (*L* ^∗^ values) in all materials except ML‐B and affected *a* ^∗^ and *b* ^∗^ values in some groups (Figure 4), generally darkening and altering the color. Despite these changes, pairwise comparisons showed consistent trends between unpolished and polished surfaces (Figure 3), suggesting polishing enhances clarity without noticeably changing the overall shade. Therefore, comparisons between unpolished and polished surfaces should be interpreted with caution. Although statistically significant differences were observed, the magnitude of these differences was small and unlikely to be clinically meaningful. This interpretation is further supported by the overall color change (Δ*E*) analysis, which revealed statistically significant differences among the six materials; however, the Δ*E* values were minimal. Moreover, brushing has been reported to increase color changes, as noted by Hickl et al. [18] and Raffaini et al. [20], so proper cleaning methods are important to maintain splint appearance.

### 4.2. Surface Roughness

The surface roughness values of unpolished occlusal splints were ranked as follows: 3D‐A > SC > HC > 3D‐B > ML‐B > ML‐A. Contrary to expectations, CAD–CAM methods did not consistently outperform conventional techniques, because their effectiveness varied with the type of CAD–CAM material [21]. In this study, ML splints exhibited the smoothest surfaces on the unpolished side, indicating that milling technology generally produces a finer surface finish. In contrast, the 3D splint showed both higher and lower Ra values compared with conventional methods. Therefore, CAD–CAM fabrication does not necessarily result in superior surface roughness, and the outcome largely depends on material properties [12]. Careful evaluation of materials is therefore essential before selecting a fabrication method.

Focusing on the polished surfaces of occlusal splints in this study, although the unpolished splints initially showed differences in surface roughness after fabrication, all materials achieved comparable surface roughness after polishing using a standardized protocol [13]. This indicates that the polishing process can effectively minimize surface irregularities across different materials [22], reducing variations caused by the fabrication method and resulting in clinically acceptable smoothness for all splints. Dentists can utilize the same polishing equipment, already available in dental clinics, for conventional and CAD–CAM splints during treatment [23].

Surface roughness data can guide material selection for dental patients, because higher surface roughness is associated with increased biofilm formation [12]. In the present study, ML splints demonstrated the lowest surface roughness, indicating superior performance in this physical property. However, the previous study reported that the fabrication method did not significantly affect the adhesion of *Candida albicans* and *Streptococcus mutans* [24], suggesting that all types of occlusal splint materials have a similar potential for microbial accumulation [25]. Nevertheless, the maximum surface roughness threshold for bacterial adhesion is considered to be 0.2 µm [26]. In the current study, only ML‐A and ML‐B splints demonstrated surface roughness values below this threshold for both unpolished and polished surfaces, indicating better performance compared to the other materials.

### 4.3. Microhardness

Microhardness, a key mechanical property of occlusal splints, reflects their durability, with higher values indicating greater longevity. Among conventional materials, HC splints demonstrated the second‐highest microhardness (17.58–18.85 VHN), while SC splints exhibited the lowest microhardness (8.67–8.75 VHN), aligning with Gibreel et al. [17] that SC had significantly lower microhardness than the HC group. Because the HC splint is made from PMMA with a higher degree of polymerization than the SC splint, it exhibited greater microhardness [7]. In contrast, SC splints may release more residual monomer, which can affect microhardness, cause dimensional shrinkage, and potentially trigger allergic reactions [27, 28]. However, SC splints are suitable for protecting against tooth wear, especially on exposed dentin [7]. Therefore, the selection of conventional materials should consider multiple properties as well as individual patient needs.

In the CAD–CAM group, ML splints showed higher microhardness than 3D splints, with ML‐B recording the highest microhardness (19.53–19.74 VHN) among the tested materials. Similarly, Gibreel et al. [29] and Berli et al. [30] found that ML materials demonstrated higher microhardness compared with other fabrication methods. Additionally, the ML splint had the highest fracture resistance compared with the other splint groups [31], further emphasizing their mechanical superiority. Conversely, 3D occlusal splints had the lowest microhardness (4.88–11.29 VHN)[32]. This is attributed to the innovative composition of 3D‐printing materials, designed to be printable and biocompatible, which compromises their microhardness compared with PMMA‐based ML materials [33]. Hence, the variations in microhardness were influenced by the fabrication method and the types of materials used, which significantly impacted the observed differences in the data [34].

The superior microhardness of the ML‐B group, followed by the HC group, suggests that only ML‐B splints are comparable to conventional splints in terms of microhardness. Although the other groups exhibited lower microhardness, they may still be appropriate for patients with severe attrition or exposed dentin, where a softer material helped reduce further tooth wear or sensitivity [35]. ML splints demonstrated the highest microhardness because they are produced from PMMA blocks that undergo controlled manufacturing processes to optimize their mechanical properties [36, 37]. These PMMA blocks offer superior resistance to scratches, wear, and occlusal load, indicating better long‐term performance in clinical applications.

The microhardness of the unpolished surfaces contributes to the splint’s rigidity and adaptability to the teeth, while polished surfaces enhance the splint’s ability to withstand heavy occlusal forces from the opposing dental arch. This study found that polishing slightly altered microhardness across most materials, with significant reductions observed only in the HC and 3D‐B groups. This suggests that the polished surface generally retains mechanical properties comparable to the unpolished material, except for these two groups, which may experience diminished resistance to occlusal forces after polishing.

### 4.4. Limitations

This study provides valuable insights into occlusal splint materials, supporting the optimization of material selection and fabrication to improve clinical outcomes. However, this study has several limitations. First, the color was only assessed and compared between different occlusal splint materials without considering aging conditions, which limits its relevance to clinical applications. Future studies should therefore examine the color stability of these materials under aging conditions. Second, the polishing protocol used was standardized and did not reflect the variety of techniques and equipment used in clinical practice. Consequently, the surface roughness observed may differ from what is typically seen in clinical settings, highlighting the need for further research under different polishing conditions. Third, the study measured the microhardness of the splint materials but did not assess their wear resistance. Because the interaction between the occlusal splint and opposing teeth was not fully captured, clinical wear behaviors may differ. Future research should explore the wear resistance and performance of these splints in functional conditions. Last, the study did not simulate long‐term use in the oral environment, where factors like saliva, temperature changes, and mechanical forces impact the splints. Further studies are needed to evaluate their long‐term performance in conditions that closely mimic the oral environment.

## 5. Conclusion

Occlusal splints fabricated using SC, HC, ML, and 3D‐printed methods exhibited significant differences in color, surface roughness, and microhardness, with variations observed between conventional and CAD–CAM fabrication techniques. Among these, the ML‐B occlusal splint demonstrated superior overall performance, with the highest *L* ^∗^ value, an average *a* ^∗^ value, a near‐zero *b* ^∗^ value, one of the lowest surface roughness values, and the highest microhardness value. Polishing significantly changed the splint’s colors following the trend *L* ^∗^ > *a* ^∗^ > *b* ^∗^, reduced surface roughness across all groups, and decreased microhardness in the HC and 3D‐B groups. These findings highlight that some CAD–CAM occlusal splint materials exhibit properties comparable to those of conventional materials. Therefore, the selection of fabrication methods should consider not only the technique but also the material itself to optimize splint performance for patients with varying oral conditions.

## Author Contributions

Uthai Uma developed ideas and a conceptual framework for the study. Uthai Uma, Thanchanit Lertyingyos, and Tharathip Lilitsuvan reviewed relevant literature. Uthai Uma designed the study, while Uthai Uma, Thanchanit Lertyingyos, and Tharathip Lilitsuvan conducted experiments and gathered data. Uthai Uma carried out the initial screening, performed statistical analysis, critically interpreted the data, and wrote the draft of the manuscript. Thanchanit Lertyingyos and Tharathip Lilitsuvan offered suggestions for the manuscript.

## Acknowledgments

The authors acknowledge the research funding provided by the Dental Research Fund at the Faculty of Dentistry, Chulalongkorn University, Bangkok, Thailand, under Grant 3200502#06/2024. We are also greatly appreciative of the crucial support from the Dental Materials Research and Development Center and the Oral Biology Research Center at the Faculty of Dentistry, Chulalongkorn University. Additionally, we wish to extend our sincere thanks to Dr. Kevin Tompkins for his meticulous review and English language editing of the manuscript.

## Funding

This work was supported by the Dental Research Fund, Dental Research Project, Faculty of Dentistry, Chulalongkorn University, Bangkok, Thailand, under Grant 3200502#06/2024.

## Disclosure

Uthai Uma, Thanchanit Lertyingyos, and Tharathip Lilitsuvan revised and approved the final version of the manuscript.

## Conflicts of Interest

The authors declare no conflicts of interest.

## Data Availability

The data that support the findings of this study are available from the corresponding author upon reasonable request.

## References

[1] Albagieh H. , Alomran I. , and Binakresh A. , et al.Occlusal Splints-Types and Effectiveness in Temporomandibular Disorder Management, The Saudi Dental Journal. (2023) 35, no. 1, 70–79, 10.1016/j.sdentj.2022.12.013. 36817028 PMC9931504

[2] Al-Moraissi E. A. , Farea R. , Qasem K. A. , Al-Wadeai M. S. , Al-Sabahi M. E. , and Al-Iryani G. M. , Effectiveness of Occlusal Splint Therapy in the Management of Temporomandibular Disorders: Network Meta-Analysis of Randomized Controlled Trials, International Journal of Oral and Maxillofacial Surgery. (2020) 49, no. 8, 1042–1056, 10.1016/j.ijom.2020.01.004. 31982236

[3] Singh B. P. , Singh N. , and Jayaraman S. , et al.Occlusal Interventions for Managing Temporomandibular Disorders, Cochrane Database of Systematic Reviews. (2024) 2024, no. 9, 10.1002/14651858.cd012850.pub2, CD012850. PMC11403706 39282765

[4] Hardy R. S. and Bonsor S. J. , The Efficacy of Occlusal Splints in the Treatment of Bruxism: A Systematic Review, Journal of Dentistry. (2021) 108, 10.1016/j.jdent.2021.103621, 103621. 33652054

[5] Uma U. , Fongpisuttikul P. , Padungpipatbawon P. , and Luyapan P. , Prevalence, Awareness, and Management of Bruxism in Thai Dental Students: A Cross-Sectional Study, Cranio: The Journal of Craniomandibular Practice. (2024) 42, no. 5, 532–538, 10.1080/08869634.2021.2015557. 34895099

[6] Crout D. K. , Anatomy of an Occlusal Splint, General Dentistry. (2017) 65, no. 2, 52–59. 28253183

[7] Nassif M. , Haddad C. , Habli L. , and Zoghby A. , Materials and Manufacturing Techniques for Occlusal Splints: A Literature Review, Journal of Oral Rehabilitation. (2023) 50, no. 11, 1348–1354, 10.1111/joor.13550. 37392157

[8] Orgev A. , Levon J. A. , Chu T.-G. , Morton D. , and Lin W.-S. , The Effects of Manufacturing Technologies on the Surface Accuracy of CAD-CAM Occlusal Splints, Journal of Prosthodontics-Implant Esthetic and Reconstructive Dentistry. (2023) 32, no. 8, 697–705, 10.1111/jopr.13610. 36227731

[9] Patzelt S. B. M. , Krugel M. , and Wesemann C. , et al.In Vitro Time Efficiency, Fit, and Wear of Conventionally-Versus Digitally-Fabricated Occlusal Splints, Materials. (2022) 15, no. 3, 10.3390/ma15031085, 1085. 35161032 PMC8837971

[10] Blasi A. , Henarejos-Domingo V. , Palacios-Bañuelos R. , Vidal-Ponsoda C. , Aparicio C. , and Roig M. , CAD-CAM and Analog Occlusal Splints Comparison Based on the Amount of Occlusal Adjustments. 3D Analysis of the Volumetric Changes: A Pilot Study, Journal of Esthetic and Restorative Dentistry. (2023) 35, no. 8, 1271–1278, 10.1111/jerd.13080. 37395327

[11] American College of Prosthodontists and ACP Education Foundation , Glossary of Digital Dental Terms, 2nd Edition, Journal of Prosthodontics. (2021) 30, no. S3, 172–181, 10.1111/jopr.13439. 34878191

[12] Wuersching S. N. , Westphal D. , Stawarczyk B. , Edelhoff D. , and Kollmuss M. , Surface Properties and Initial Bacterial Biofilm Growth on 3D-Printed Oral Appliances: A Comparative In Vitro Study, Clinical Oral Investigations. (2023) 27, no. 6, 2667–2677, 10.1007/s00784-022-04838-7. 36576565 PMC10264496

[13] Guimaraes D. M. , Campaner M. , Santos R. W. D. , Pesqueira A. A. , and Medeiros R. A. , Evaluation of the Mechanical Properties of Different Materials for Manufacturing Occlusal Splints, Brazilian Oral Research. (2023) 37, 10.1590/1807-3107bor-2023.vol37.0034, e034. 37132723

[14] Perea-Lowery L. , Gibreel M. , Vallittu P. K. , and Lassila L. , Evaluation of the Mechanical Properties and Degree of Conversion of 3D Printed Splint Material, Journal of the Mechanical Behavior of Biomedical Materials. (2021) 115, 10.1016/j.jmbbm.2020.104254, 104254. 33333480

[15] Carter E. C. , Schanda J. , and Hirschler R. , et al.Colorimetry: Technical Report, 4th ed, 2018, Internaitonal Commission on Illumination.

[16] Layton D. M. , Morgano S. M. , and Muller F. , et al.The Glossary of Prosthodontic Terms 2023: Tenth Edition, Journal of Prosthetic Dentistry. (2023) 130, no. 4 Suppl 1, e1–e3, 10.1016/j.prosdent.2023.03.003. 37914441

[17] Gibreel M. , Perea-Lowery L. , Vallittu P. K. , and Lassila L. , Characterization of Occlusal Splint Materials: CAD-CAM Versus Conventional Resins, Journal of the Mechanical Behavior of Biomedical Materials. (2021) 124, 10.1016/j.jmbbm.2021.104813, 104813. 34530298

[18] Hickl V. , Strasser T. , Schmid A. , and Rosentritt M. , Effects of Storage and Toothbrush Simulation on Color, Gloss, and Roughness of CAD/CAM, Hand-Cast, Thermoforming, and 3D-Printed Splint Materials, Clinical Oral Investigations. (2022) 26, no. 5, 4183–4194, 10.1007/s00784-022-04391-3. 35119536 PMC9072518

[19] Dylina T. J. , A Common-Sense Approach to Splint Therapy, The Journal of Prosthetic Dentistry. (2001) 86, no. 5, 539–545, 10.1067/mpr.2001.118878, 2-s2.0-0035514043. 11725283

[20] Raffaini J. C. , Soares E. J. , and Oliveira R. F. L. , et al.Effect of Artificial Aging on Mechanical and Physical Properties of CAD-CAM PMMA Resins for Occlusal Splints, The Journal of Advanced Prosthodontics. (2023) 15, no. 5, 227–237, 10.4047/jap.2023.15.5.227. 37936836 PMC10625884

[21] Maleki T. , Meinen J. , Coldea A. , Reymus M. , Edelhoff D. , and Stawarczyk B. , Mechanical and Physical Properties of Splint Materials for Oral Appliances Produced by Additive, Subtractive and Conventional Manufacturing, Dental Materials. (2024) 40, no. 8, 1171–1183, 10.1016/j.dental.2024.05.030. 38851965

[22] Uma U. , Chansri J. , Itthipornpaisan T. , and Thahong T. , Surface Roughness and Microhardness of Heat-Cured, Milled, and 3D-Printed Occlusal Splints: A Comparative Study of Unpolished and Polished Surfaces, Natural and Life Sciences Communications. (2025) 24, no. 3, 10.12982/NLSC.2025.041, E2025041.

[23] Grymak A. , Aarts J. M. , Ma S. , Waddell J. N. , and Choi J. J. E. , Comparison of Hardness and Polishability of Various Occlusal Splint Materials, Journal of the Mechanical Behavior of Biomedical Materials. (2021) 115, 10.1016/j.jmbbm.2020.104270, 104270. 33341739

[24] Schubert A. , Bürgers R. , Baum F. , Kurbad O. , and Wassmann T. , Influence of the Manufacturing Method on the Adhesion of *Candida albicans* and *Streptococcus mutans* to Oral Splint Resins, Polymers (Basel). (2021) 13, no. 10, 10.3390/polym13101534, 1534. 34064561 PMC8150722

[25] Özarslan M. , Avcioglu N. H. , Bilgili Can D. , and Çalışkan A. , Biofilm Formation of *C. albicans* on Occlusal Device Materials and Antibiofilm Effects of Chitosan and Eugenol, The Journal of Prosthetic Dentistry. (2024) 131, no. 1, 144.e1–144.e9, 10.1016/j.prosdent.2023.10.005. 38167132

[26] Bollenl C. M. L. , Lambrechts P. , and Quirynen M. , Comparison of Surface Roughness of Oral Hard Materials to the Threshold Surface Roughness for Bacterial Plaque Retention: A Review of the Literature, Dental Materials. (1997) 13, no. 4, 258–269, 10.1016/S0109-5641(97)80038-3. 11696906

[27] Engler M. L. P. D. , Güth J.-F. , Keul C. , Erdelt K. , Edelhoff D. , and Liebermann A. , Residual Monomer Elution From Different Conventional and CAD/CAM Dental Polymers During Artificial Aging, Clinical Oral Investigations. (2020) 24, no. 1, 277–284, 10.1007/s00784-019-02947-4, 2-s2.0-85066049623. 31098712

[28] Wei X. , Pan Y. , and Wang M. , et al.Comparative Analysis of Leaching Residual Monomer and Biological Effects of Four Types of Conventional and CAD/CAM Dental Polymers: An In Vitro Study, Clinical Oral Investigations. (2022) 26, no. 3, 2887–2898, 10.1007/s00784-021-04271-2. 35083585

[29] Gibreel M. , Perea-Lowery L. , Vallittu P. K. , Garoushi S. , and Lassila L. , Two-Body Wear and Surface Hardness of Occlusal Splint Materials, Dental Materials Journal. (2022) 41, no. 6, 916–922, 10.4012/dmj.2022-100. 36288940

[30] Berli C. , Thieringer F. M. , and Sharma N. , et al.Comparing the Mechanical Properties of Pressed, Milled, and 3D-Printed Resins for Occlusal Devices, The Journal of Prosthetic Dentistry. (2020) 124, no. 6, 780–786, 10.1016/j.prosdent.2019.10.024. 31955837

[31] Abad-Coronel C. , Ruano Espinosa C. , Ordóñez Palacios S. , Paltán C. A. , and Fajardo J. I. , Comparative Analysis Between Conventional Acrylic, CAD/CAM Milled, and 3D CAD/CAM Printed Occlusal Splints, Materials. (2023) 16, no. 18, 10.3390/ma16186269, 6269. 37763547 PMC10532716

[32] Prpic V. , Spehar F. , Stajdohar D. , Bjelica R. , Cimic S. , and Par M. , Mechanical Properties of 3D-Printed Occlusal Splint Materials, Dentistry Journal. (2023) 11, no. 8, 10.3390/dj11080199, 199. 37623295 PMC10453325

[33] de Paula Lopez V. , Dias Corpa Tardelli J. , Botelho A. L. , Marcondes Agnelli J. A. , and Cândido dos Reis A. , Mechanical Performance of 3-Dimensionally Printed Resins Compared With Conventional and Milled Resins for the Manufacture of Occlusal Devices: A Systematic Review, The Journal of Prosthetic Dentistry. (2024) 132, no. 6, 1262–1269, 10.1016/j.prosdent.2022.12.006. 36631367

[34] Prpic V. , Slacanin I. , Schauperl Z. , Catic A. , Dulcic N. , and Cimic S. , A Study of the Flexural Strength and Surface Hardness of Different Materials and Technologies for Occlusal Device Fabrication, The Journal of Prosthetic Dentistry. (2019) 121, no. 6, 955–959, 10.1016/j.prosdent.2018.09.022, 2-s2.0-85060740269. 30711296

[35] Potewiratnanond P. , Ekrojanakul C. , Harikul T. , and Kositvanich R. , Wear Effects Between Polymethyl Methacrylate Occlusal Splints and Opposing Dentin Surfaces During Bruxism Mimicking Events, BDJ Open. (2023) 9, no. 1, 10.1038/s41405-023-00148-6, 21. 37301831 PMC10257649

[36] Benli M. , Al-Haj Husain N. , and Ozcan M. , Mechanical and Chemical Characterization of Contemporary Occlusal Splint Materials Fabricated With Different Methods: A Systematic Review, Clinical Oral Investigations. (2023) 27, no. 12, 7115–7141, 10.1007/s00784-023-05360-0. 37910242

[37] Valenti C. , Federici M. I. , and Coniglio M. , et al.Mechanical and Biological Properties of Polymer Materials for Oral Appliances Produced With Additive 3D Printing and Subtractive CAD-CAM Techniques Compared to Conventional Methods: A Systematic Review and Meta-Analysis, Clinical Oral Investigations. (2024) 28, no. 7, 10.1007/s00784-024-05772-6, 396. 38916682

